# The influence of hospital volume and physician volume on early mortality in acute promyelocytic leukemia patients

**DOI:** 10.1007/s00277-024-05616-z

**Published:** 2024-03-26

**Authors:** Chia-Ying Wu, Chiu-Mei Yeh, Chun-Kuang Tsai, Chia-Jen Liu

**Affiliations:** 1 Department of Medicine, Taipei Veterans General Hospital, Taipei, Taiwan; 2 Division of Hematology, Department of Medicine, Taipei Veterans General Hospital, Taipei, Taiwan; 3 Institute of Public Health, National Yang Ming Chiao Tung University, Taipei, Taiwan; 4 School of Medicine, National Yang Ming Chiao Tung University, Taipei, Taiwan; 5 Division of Hematology, Institute of Clinical Medicine, National Yang Ming Chiao Tung University, Taipei Veterans General Hospital, Taipei, Taiwan; 6 Division of Hematology, Institute of Emergency and Critical Care Medicine, National Yang Ming Chiao Tung University, Taipei Veterans General Hospital, Taipei, Taiwan

**Keywords:** Acute promyelocytic leukemia, Physician volume, Hospital volume, Early mortality

## Abstract

Acute promyelocytic leukemia (APL) is a highly curable hematologic malignancy in the era of all-trans retinoic acid (ATRA) combination treatment. However, only a modest change in early mortality rate has been observed despite the wide availability of ATRA. In addition to the clinical characteristics of APL patients, studies on the hospital volume-outcome relationship and the physician volume-outcome relationship remained limited. We aim to evaluate the association between hospital and physician volume and the early mortality rate among APL patients. The patients were collected from Taiwan’s National Health Insurance Research Database (NHIRD). Early mortality is defined as death within 30 days of diagnosis. Patients were categorized into four groups according to individual cumulative hospital and physician volume. The risk of all-cause mortality in APL patients with different cumulative volume groups was compared using a Cox proportional hazard model. The probability of overall survival was estimated using the Kaplan–Meier method. All 741 patients were divided into four quartile volume groups. In the multivariate analysis, only physician volume was significantly associated with early mortality rate. The physician volume of the highest quartile was a protective factor for early mortality compared with the physician volume of the lowest quartile (HR 0.10, 95% CI 0.02–0.65). Hospital characteristics were not associated with early mortality. In the sensitivity analyses, the results remained consistent using two other different definitions of early mortality. Higher physician volume was independently associated with lower early mortality, while hospital volume was not. Enhancing the clinical expertise of low-volume physicians may ensure better outcomes.

**Supplementary Information:**

The online version contains supplementary material available at 10.1007/s00277-024-05616-z.

## Introduction

Acute promyelocytic leukemia (APL) is a subtype of acute myeloid leukemia (AML) accounting for 10 to 15% of newly diagnosed AML cases annually [1]. The specific chromosomal translocation is characterized by a translocation between the promyelocytic leukemia (PML) gene on chromosome 15 and the retinoic acid receptor α (RARA) gene on chromosome 17 [2]. The disease is characterized by severe coagulopathy, causing fatal hemorrhagic complications. In the mid-1990s, the revolutionized all-trans retinoic acid (ATRA) differentiation therapy [3–6] significantly improved 30-day early mortality by ameliorating coagulopathy and bleeding events from 26% (1988–1995) to 14% (2004–2011) [7, 8]. Since then, APL has evolved from a rapidly fatal disease to a highly curable condition with a cure rate exceeding 80% [9].

Over the past several decades, the mortality rate in the first 4–6 weeks has been reported to be less than 5% in well-designed clinical trials [10]. However, these patients were carefully selected and might not reflect the real-world situation [11]. In population-based analyses, the early mortality rate remains high (29%, median 4 days) [12]. The causes of death in these patients include delayed disease identification and lack of initial proactive treatment, leading to fatal bleeding events [13, 14]. A study reported that induction mortality was 9% among 732 patients. The major categories of mortality in induction failure were primarily hemorrhage death (5%), followed by infection (2.3%), and differentiation syndrome (1.4%) [15]. The life-threatening coagulopathy before and during induction therapy has always been the major concern of treatment failure among APL patients [1, 16, 17].

While most studies have focused on the association between clinical features and survival rate, few studies have examined the volume of treatment facilities or the effect of physician volume. Intuitively, medical centers with higher volumes were more likely to see better prognosis [18, 19]. However, previous studies usually used hospital volume to speculate on physician volume indirectly. With APL patients, the physician’s ability to recognize the disease and initiate ATRA timely is regarded as the key factor in patient outcome [20, 21]. There are also limited studies investigating the independent role of physicians in treating APL and its association with early mortality. To fill this knowledge gap, we performed a nationwide population-based study to identify the impact of hospital volume and physician volume on real-world APL patients.

## Materials and methods

### Data source

This is a nationwide population-based retrospective cohort study. We collected data from the National Health Insurance Research Database (NHIRD). Initiated on March 1, 1995, NHIRD served as Taiwan’s single-payer mandatory insurance system. The database of this program covers more than 99.9% of the population in Taiwan. To protect patient confidentiality, data were retrieved and analyzed by on-site analysis at the Health and Wellness Data Science Center via remote connection to the Ministry of Health and Welfare server. The NHIRD contains information on the demographic characteristics of hospitals and physicians, ambulatory care, admissions, procedures, diagnoses, and prescribed medications. The diagnosis coding system was used following the International Classification of Diseases (ICD) revision 9th and 10th system to classify diagnostic, health services utilization, and death data.

### Study population

Patients enrolled in this study were newly diagnosed with APL and registered in the Registry for Catastrophic Illness Patients (RCIP) between January 1, 2000, and December 31, 2017, in Taiwan. RCIP includes people who have severe diseases, including cancer, and they receive a co-payment waiver under the NHI program by using ICD-9-CM codes 205–207. Not included are 205.1 (chronic myeloid leukemia), 206.1 (chronic monocytic leukemia), and 207.1 (chronic erythremia), and ICD-10-CM codes C92–C94 (not including C92.1, C93.1 [chronic myelomonocytic leukemia], and C94.1 [chronic erythremia]). Furthermore, the enrolled patients should receive ATRA or ATO for more than 1 week after diagnosis to avoid coding errors. Patients diagnosed at age < 20 or with antecedent cancer before the diagnosis of APL were excluded.

### Variables

The primary endpoint of the study was 30-day mortality. The information on the date and cause of death is contained in the National Cause of Death Data. We define the cumulative physician volume as the total number of APL patients treated by each hematologist before treating the index patient. The definition of cumulative hospital volume is the total number of APL patients treated in this hospital right before treating the index patient. Each patient’s provider could have had different values of physician volume and hospital volume. According to the cumulative numbers of physicians and hospitals, all patients were stratified into four quartiles: lowest, middle-low, middle-high, and highest. Patient information such as age, sex, comorbidities (hypertension, diabetes mellitus, dyslipidemia, cerebrovascular accident, coronary artery disease, chronic kidney disease, and bleeding history), urbanization, and socioeconomic status were analyzed.

Furthermore, provider baseline characteristics such as hospital ownership, hospital region, accreditation level of hospital, physician age, physician sex, and physician experience were also included in the analysis.

### Statistical analysis

The categorical variables were expressed as counts and proportions. We performed Pearson’s chi-squared test or Fisher’s exact test to analyze the differences between categorical variables, while the Mann–Whitney *U* test was used for continuous variables. The probability of overall survival (OS) was measured using the Kaplan-Meier method from the time of diagnosis to death or last follow-up. A log-rank test provided additional estimates of the group differences. Cox proportional hazard models were constructed to determine whether there were significant differences in all-cause mortality risk between different patients’ cumulative volume groups. In the multivariate analysis, we used the frailty model for Cox regression to adjust for physician-level random effects. Hazard ratios (HRs) and 95% confidence intervals (CIs) were calculated for risk factors.

Early mortality was defined as death within 30 days after APL diagnosis. We performed a sensitivity analysis to assess the influence of different definitions of early mortality, which were 60- and 90-day mortality, and adjusted for patient and physician characteristics. All statistical tests were two-sided, and the significance level was set at 0.05. All data were analyzed with SAS 9.4 software (SAS Institute Inc., Cary, NC) and STATA statistical software, version 15.1 (StataCorp, College Station, TX). The present study was approved by the Institutional Review Board at Taipei Veterans General Hospital (no. 2019-07-054BC).

## Results

### Clinical characteristics of the study population

**Fig. 1:**
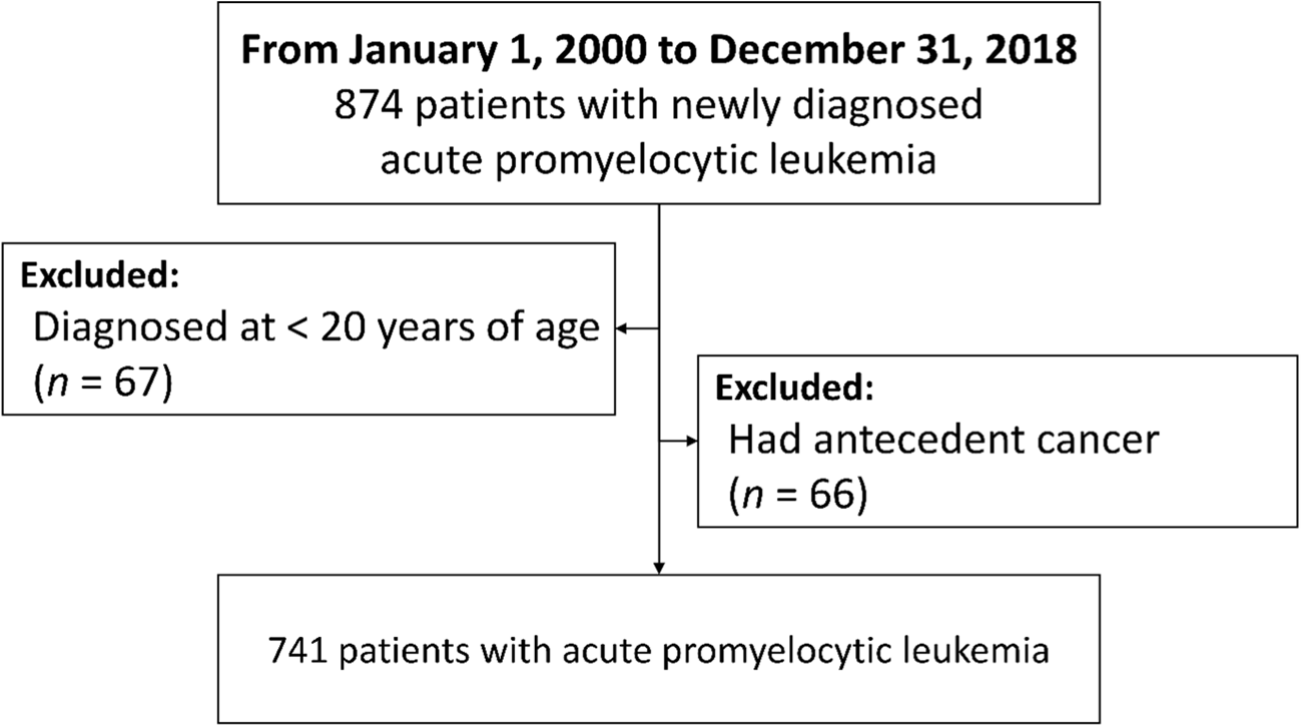
Patient selection flow chart

Our study cohort included 874 patients with newly diagnosed APL between January 1, 2000, and December 31, 2018, a 19-year time span. Patients younger than age 20 (*n* = 67) or those with antecedent cancer (*n* = 66) were excluded. A total of 741 patients with APL were eligible for the study (Fig. 1).

The patients’ baseline characteristics are shown in Table 1. The median age at diagnosis was 47 (range 20 to 88). A total of 429 patients (57.9%) were less than 50 years of age, and 53.6% were male. The majority of patients were treated in medical centers (88.5%). The median cumulative volume was 8 (IQR 4–13) for physicians and 30 (IQR 13–58) for hospitals. A total of 187 physicians included for analysis were classified into four degrees of volume, including lowest, middle-low, middle-high, and highest. Patients treated by physicians with higher physician volume were more likely to be treated in medical centers (75.4%, 86.0%, 94.9%, and 96.6%, respectively, from the first to fourth quartile, *p* < 0.001). Higher physician-volume groups were more likely to be treated by physicians over 45 years old (32.8%, 35.5%, 44.4%, and 51.9%, respectively, from the first to fourth quartile, *p* < 0.001). The treatment of this cohort involved 364 patients (49.1%) treated with physicians whose experience was ≥ 5 years. Patients in the higher physician-volume group were more likely to be treated by experienced (≥ 5 years) physicians (4.4%, 27.3%, 67.4%, and 90.9%, respectively, from the 1st to the 4th quartile, *p* < 0.001). There were only eight patients in the lowest physician volume group (4.4% of 183 patients) treated by experienced physicians, 47 patients in the middle-low physician volume group (27.3% of 172 patients), 120 patients in the middle-high physician volume group (67.4% of 178), and 189 patients in the highest physician volume group (90.9% of 208).

**Table 1:** Baseline characteristics of patients with acute promyelocytic leukemia

Characteristics	Total, *n* = 741	Physician volume				*p* value
		Lowest*, n* = 183	Middle-low*, n* = 172	Middle-high*, n* = 178	Highest*, n* = 208	
Median age, years (range)	47 (20–88)	48 (21–82)	46 (20–81)	49 (20–82)	44 (21–88)	0.143
Age, years						
≥ 50	312 (42.1)	79 (43.2)	67 (39.0)	82 (46.1)	84 (40.4)	0.535
< 50	429 (57.9)	104 (56.8)	105 (61.0)	96 (53.9)	124 (59.6)	
Sex						
Male	397 (53.6)	100 (54.6)	85 (49.4)	98 (55.1)	114 (54.8)	0.668
Female	344 (46.4)	83 (45.4)	87 (50.6)	80 (44.9)	94 (45.2)	
Comorbidities						
Hypertension	248 (33.5)	55 (30.1)	51 (29.7)	71 (39.9)	71 (34.1)	0.144
Diabetes mellitus	192 (25.9)	45 (24.6)	41 (23.8)	45 (25.3)	61 (29.3)	0.604
Dyslipidemia	228 (30.8)	42 (23.0)	50 (29.1)	57 (32.0)	79 (38.0)	0.014
Cerebrovascular accident	103 (13.9)	22 (12.0)	28 (16.3)	22 (12.4)	31 (14.9)	0.597
Coronary artery disease	138 (18.6)	34 (18.6)	29 (16.9)	34 (19.1)	41 (19.7)	0.910
Chronic kidney disease	91 (12.3)	24 (13.1)	18 (10.5)	20 (11.2)	29 (13.9)	0.715
Bleeding history	316 (42.6)	70 (38.3)	76 (44.2)	76 (42.7)	94 (45.2)	0.539
Degree of urbanization						
Urban	422 (57.0)	106 (57.9)	93 (54.1)	95 (53.4)	128 (61.5)	0.673
Suburban	206 (27.8)	54 (29.5)	47 (27.3)	52 (29.2)	53 (25.5)	
Rural	69 (9.3)	13 (7.1)	18 (10.5)	20 (11.2)	18 (8.7)	
Unknown	44 (5.9)	10 (5.5)	14 (8.1)	11 (6.2)	9 (4.3)	
Income level						
Low income	449 (60.6)	113 (61.7)	114 (66.3)	100 (56.2)	122 (58.7)	0.437
Median income	177 (23.9)	43 (23.5)	34 (19.8)	49 (27.5)	51 (24.5)	
High income	102 (13.8)	24 (13.1)	19 (11.0)	25 (14.0)	34 (16.3)	
Hospital ownership						
Private	445 (60.1)	119 (65.0)	105 (61.0)	100 (56.2)	121 (58.2)	0.337
Public	296 (39.9)	64 (35.0)	67 (39.0)	78 (43.8)	87 (41.8)	
Hospital region						
North	364 (49.1)	99 (54.1)	82 (47.7)	85 (47.8)	98 (47.1)	< 0.001
Middle	240 (32.4)	61 (33.3)	61 (35.5)	58 (32.6)	60 (28.8)	
South + East	137 (18.5)	23 (12.6)	29 (16.9)	35 (19.7)	50 (24.0)	
Medical center status						
Non-medical center	85 (11.5)	45 (24.6)	24 (14.0)	9 (5.1)	7 (3.4)	< 0.001
Medical center	656 (88.5)	138 (75.4)	148 (86.0)	169 (94.9)	201 (96.6)	
Physician age						
< 45	433 (58.4)	123 (67.2)	111 (64.5)	99 (55.6)	100 (48.1)	< 0.001
≥ 45	308 (41.6)	60 (32.8)	61 (35.5)	79 (44.4)	108 (51.9)	
Physician sex						
Male	641 (86.5)	159 (86.9)	150 (87.2)	158 (88.8)	174 (83.7)	0.508
Female	100 (13.5)	24 (13.1)	22 (12.8)	20 (11.2)	34 (16.3)	
Physician experience ≥ 5 years	364 (49.1)	8 (4.4)	47 (27.3)	120 (67.4)	189 (90.9)	< 0.001

*IQR* interquartile range

Higher hospital volume tended to have a higher proportion of public ownership (26.6%, 35.2%, 42.6%, and 54.8%, respectively, from the first to fourth quartile) (Supplementary Table 1). There was also a higher percentage of patients treated in medical centers with higher hospital volume (62%, 90.7%, 100%, and 100%, respectively, from the first to the fourth quartile). All the patients in the middle-high and the highest hospital volume were treated in medical centers. The characteristics of patients treated by the lowest hospital volume had fewer risks of dyslipidemia, but other comorbidities were similar among all volumes of hospitals. Higher-volume hospitals tended to have more patients treated by experienced physicians (16.4%, 38.9%, 67.2%, and 72.9%, respectively, from the first to fourth quartile).

### Overall survival and risk factors of mortality

**Fig. 2:**
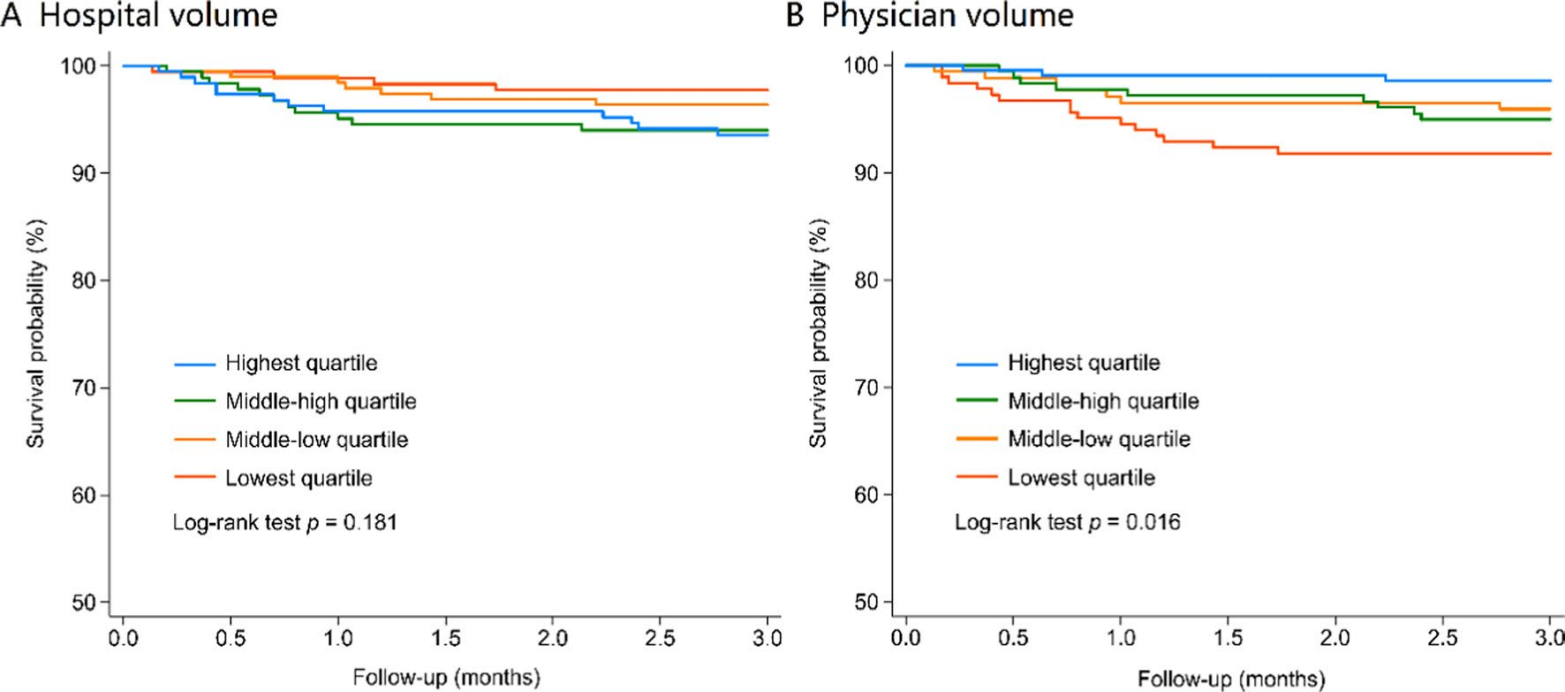
Three-month survival between patients for acute promyelocytic leukemia divided by hospital and physician

In univariate analysis, the highest quartile physician volume was a significant protective factor for 30-day early mortality (HR 0.17, 95% CI 0.04–0.79; *p* = 0.023). In contrast, hospital volume, location, and medical center status were not associated with early mortality. In the multivariate analysis, after adjusting for patient and physician characteristics, patients treated by the highest physician volume saw a protective factor in 30-day mortality (HR 0.10, 95% CI 0.02–0.65) (Table 2). In the sensitivity analysis, we used two other alternative follow-up durations of mortality, which were 60- and 90-day mortality. The findings for these durations were comparable to those for 30-day mortality, with adjusted HR 0.06 (95% CI 0.01–0.39; *p* = 0.003) in 60-day mortality and adjusted HR 0.08 (95% CI 0.02–0.37; *p* = 0.001) in 90-day mortality, respectively, in Table 3. When examining the long-term effect over a period of 5 years, the middle-high-volume physician group did not exhibit a significant reduction in 30- or 90-day early mortality. However, they did show improved long-term outcomes, with reduced 5-year mortality rates (adjusted HR 0.48, 95% CI 0.26–0.90; *p* = 0.021). The highest physician volume was a significantly predictor of 5-year survival (adjusted HR 0.27, 95% CI 0.13–0.58; *p* = 0.001). The Kaplan–Meier curves show that patients in the higher physician volume group had significantly better 3-month overall survival (log-rank test *p* = 0.016, Fig. 2B), while there was no survival difference between patients in different hospital volume groups (log-rank test *p* = 0.181, Fig. 2A).

**Table 2:** Risk factors for early mortality (30-day) for acute promyelocytic leukemia patients

Predictive variables	Univariate analysis		Multivariate analysis^a^	
	HR (95% CI)	*p* value	HR (95% CI)	*p* value
Age ≥ 50	4.74 (1.75–12.85)	0.002	2.71 (0.83–8.80)	0.098
Sex (male)	2.99 (1.10–8.09)	0.032	1.98 (0.69–5.70)	0.204
Comorbidities				
Hypertension	2.42 (1.04–5.59)	0.039	0.65 (0.22–1.96)	0.444
Diabetes mellitus	2.40 (1.04–5.56)	0.041	0.98 (0.35–2.73)	0.967
Dyslipidemia	3.30 (1.41–7.72)	0.006	2.09 (0.71–6.15)	0.181
Cerebrovascular accident	3.65 (1.53–8.69)	0.004	2.20 (0.83–5.85)	0.113
Coronary artery disease	3.08 (1.32–7.20)	0.010	1.11 (0.39–3.18)	0.842
Chronic kidney disease	3.40 (1.38–8.33)	0.008	2.02 (0.70–5.81)	0.193
Bleeding history	1.95 (0.83–4.56)	0.124	1.25 (0.48–3.29)	0.646
Degree of urbanization				
Urban	Reference		Reference	
Suburban	1.02 (0.38–2.71)	0.974	0.83 (0.30–2.30)	0.719
Rural	1.01 (0.23–4.51)	0.991	1.01 (0.21–4.85)	0.986
Income level				
Low income	Reference		Reference	
Median income	0.58 (0.17–2.04)	0.397	0.60 (0.16–2.23)	0.445
High income	1.71 (0.61–4.79)	0.310	1.33 (0.44–4.05)	0.615
Hospital volume				
Lowest quartile	Reference			
Middle-low quartile	1.38 (0.23–8.23)	0.727		
Middle-high quartile	4.41 (0.95–20.40)	0.058		
Highest quartile	3.82 (0.81–17.99)	0.090		
Hospital ownership				
Private	Reference			
Public	1.05 (0.45–2.45)	0.917		
Hospital region				
North	Reference			
Middle	1.91 (0.75–4.84)	0.173		
South	1.54 (0.46–5.10)	0.484		
East	–			
Medical center status				
Non-medical center	Reference			
Medical center	1.30 (0.30–5.56)	0.724		
Physician volume				
Lowest quartile	Reference		Reference	
Middle-low quartile	0.63 (0.23–1.73)	0.370	0.55 (0.18–1.69)	0.295
Middle-high quartile	0.40 (0.13–1.29)	0.126	0.30 (0.07–1.31)	0.109
Highest quartile	0.17 (0.04–0.79)	0.023	0.10 (0.02–0.65)	0.016
Physician age				
< 45	Reference		Reference	
≥ 45	1.17 (0.50–2.70)	0.717	1.06 (0.43–2.63)	0.903
Physician sex				
Male	1.57 (0.37–6.73)	0.541	1.38 (0.31–6.20)	0.677
Female	Reference		Reference	
Physician experience ≥ 5 years	0.59 (0.25–1.40)	0.229	1.65 (0.45–6.05)	0.448

*HR* hazard ratio, *CI* confidence interval

**Table 3:** Sensitivity analysis (30-, 60- and 90-day mortality)

Predictive variables	Univariate analysis		Multivariate analysis^a^	
	HR (95% CI)	*p* value	HR (95% CI)	*p* value
30-day mortality				
Physician volume				
Lowest quartile	Reference		Reference	
Middle-low quartile	0.63 (0.23–1.73)	0.370	0.55 (0.18–1.69)	0.295
Middle-high quartile	0.40 (0.13–1.29)	0.126	0.30 (0.07–1.31)	0.109
Highest quartile	0.17 (0.04–0.79)	0.023	0.10 (0.02–0.65)	0.016
Hospital volume				
Lowest quartile	Reference			
Middle-low quartile	1.38 (0.23–8.23)	0.727		
Middle-high quartile	4.41 (0.95–20.40)	0.058		
Highest quartile	3.82 (0.81–17.99)	0.090		
60-day mortality				
Physician volume				
Lowest quartile	Reference		Reference	
Middle-low quartile	0.42 (0.16–1.08)	0.072	0.35 (0.12–1.00)	0.051
Middle-high quartile	0.34 (0.12–0.92)	0.034	0.27 (0.07–1.00)	0.051
Highest quartile	0.11 (0.03–0.50)	0.004	0.06 (0.01–0.39)	0.003
Hospital volume				
Lowest quartile	Reference			
Middle-low quartile	1.38 (0.39–4.89)	0.619		
Middle-high quartile	2.46 (0.77–7.86)	0.127		
Highest quartile	1.92 (0.58–6.38)	0.286		
90-day mortality				
Physician volume				
Lowest quartile	Reference		Reference	
Middle-low quartile	0.49 (0.20–1.19)	0.116	0.38 (0.14–1.02)	0.055
Middle-high quartile	0.60 (0.26–1.37)	0.227	0.41 (0.13–1.27)	0.122
Highest quartile	0.17 (0.05–0.59)	0.005	0.08 (0.02–0.37)	0.001
Hospital volume				
Lowest quartile	Reference			
Middle-low quartile	1.61 (0.47–5.51)	0.446		
Middle-high quartile	2.72 (0.87–8.54)	0.087		
Highest quartile	2.90 (0.93–8.98)	0.065		
5-year mortality				
Physician volume				
Lowest quartile	Reference		Reference	
Middle-low quartile	0.91 (0.58–1.42)	0.685	0.76 (0.46–1.27)	0.298
Middle-high quartile	0.68 (0.43–1.07)	0.096	0.48 (0.26–0.90)	0.021
Highest quartile	0.44 (0.25–0.77)	0.004	0.27 (0.13–0.58)	0.001

*HR* hazard ratio, *CI* confidence interval

^a^The model was adjusted for patient and physician characteristics in the Cox multivariate analysis

## Discussion

In this nationwide population-based cohort, we investigated the early mortality of newly diagnosed APL patients who received therapy in different hospitals and with different physician volumes. Our study reveals that patients in the higher-physician volume group, not hospital volume, were associated with reduced early mortality. Consistent with the initial discovery that higher physician volume was independently associated with lower early mortality. The effects persisted across the early and 5-year endpoints. These results emphasize that the physician’s role appears to have both short- and long-term benefits for acute promyelocytic leukemia patients in APL treatment.

We included 741 patients with newly diagnosed APL in the study. In Table 1, it can be seen that a relatively higher portion of patients were treated in medical centers (88.5%), and only 11.5% of the patients were treated in non-medical centers. In Taiwan, which is affected by the national medical system, patients can get treatment in medical centers without referral and have good healthcare accessibility [22]. Therefore, most patients in Taiwan were treated in medical centers (67–84%) [23]. On the contrary, studies in Western countries investigating the association between care location and hematological malignancies have shown that more patients were treated in community hospitals (75%) than medical centers (25%) [24]. The disparate conditions may make it hard to clarify the impact between facilities’ resources and physicians’ experiences. In our study, the cumulative hospital volume was not associated with early mortality. This was different from previous literature’s findings. Ho et al.’s study noted that AML patients who were treated in high-volume designated cancer centers had a 53% reduction in the odds of death compared to those treated in low-volume hospitals (OR 0.46, CI 0.40–0.54) at 60 days of diagnosis [24]. Another study on APL reported that patients who were treated at academic medical centers had lower 30-day mortality (22% vs. 25%, *p* = 0.03). However, the explanation was attributed to experts’ appropriate initial care in the early stages of APL [25].

APL is one of the most highly curable cancers, with a complete remission rate of 80–90%. The risk of early death ranges from 17.3 to 29% in population-based analyses [12, 26]. Previous studies have reported that the differences in early death rates between medical providers might indicate delayed diagnosis and inadequate access to care [26, 27]. As shown in Table 2, we found that the highest physician volume demonstrates a negative association with early mortality rates. Experienced physicians are highly vigilant in recognizing the disease and managing early complications. Similar to those with acute coronary syndrome (ACS), clinical outcomes can be dramatically improved once ACS has been identified and treated with early intervention [28]. For patients undergoing primary percutaneous coronary intervention (PCI), the risk-adjusted mortality rate for high-volume physicians was 3.8% versus 6.5% for low-volume physicians (OR 0.58, 95% CI 0.39–0.86) [28, 29]. Similarly, with well-trained clinicians, the APL outcome discrepancy may become less evident between medical providers [30, 31].

In Fig. 2, survival in APL patients and physician volumes appear to stabilize 2 months after the initial diagnosis. Similar results were found in studies examining outcomes in APL patients. They found that survival declined sharply in the first 2 months after APL diagnosis due to hemorrhage complications but declined at a much lower rate afterward [26]. Early mortality continues to be a primary reason for decreased survival probability [13].

Historically, the physician-outcome relationship has been investigated in several diseases. A study found increased 1-year mortality rates in low-volume physician groups among heart failure patients (HR 8.64, 95% CI 2.07–36.0)[32]. With gastric cancer, a higher surgeon volume was associated with a lower 30-day mortality rate (OR 0.94, 95% CI 0.90–0.97) [33]. With colorectal cancer, however, with differences in the volume thresholds, benefits due to physicians have been inconsistently observed [34]. With hematologic malignancies, limited studies have investigated the physician-outcome relationship. Common pitfalls of the previous studies, including lack of comprehensive adjustment and physician volume, were not analyzed independently and might confound with hospital volume [24, 35, 36]. Our study, so far, is the first to demonstrate the independent physician volume effect on APL patients’ early mortality rate. The results are further consistently confirmed in different definitions of early mortality.

This study had several limitations. Due to data constraints, omitted variables might likely be another source of bias. Certain clinical information, such as white blood cell count, disseminated intravascular coagulation profile, treatment response, as well as other behavioral variables like body mass index and smoking history, were not included in our analysis as controlled variables. However, we had considered all relevant factors that could potentially confound the results with a comprehensive multivariable analysis. Second, our study lacked cytogenetic and mutation data. Therefore, in addition to the ICD diagnosis code, we required patients to receive at least 1 week of ATRA or ATO for inclusion to avoid coding errors. Third, inevitably, in the retrospective study design, selection bias could occur and might be related to the outcome. However, to the best of our knowledge, this study remains the first Asian population-based study to show real-world outcomes of APL patients.

In summary, physician volume independently improves APL patient outcomes by reducing early mortality, while hospital volume does not. These results highlight that applying quality improvement and physician training can be essential to improving APL treatment outcomes.

### Supplementary information

ESM 1 (DOCX 26 kb)
