# Supplementary material for: The influence of hospital volume and physician volume on early mortality in acute promyelocytic leukemia patients
Source: Ann Hematol. 2024 Mar 26;103(5):1577–86. doi: 10.1007/s00277-024-05616-z (PMC11009737; doi:10.1007/s00277-024-05616-z)
Supplement: Supplementary file 1 — (DOCX 26 kb) [file 277_2024_5616_MOESM1_ESM.docx]

**Supplemental Table 1. Baseline characteristics of patients with acute promyelocytic leukemia**

| **Characteristics** | **Total**  ***n* = 741** | **Hospital volume** | | | | ***p* value** |
| --- | --- | --- | --- | --- | --- | --- |
|  |  | **Lowest**  ***n* = 177** | **Middle-low**  ***n* = 193** | **Middle-high**  ***n* = 183** | **Highest**  ***n* = 188** |  |
| Median age, years (range) | 47 (20–88) | 46 (21–79) | 47 (22–88) | 47 (20–82) | 47 (21–87) | 0.449 |
| Age, years |  |  |  |  |  |  |
| ≥ 50 | 312 (42.1) | 64 (36.2) | 82 (42.5) | 81 (44.3) | 85 (45.2) | 0.299 |
| < 50 | 429 (57.9) | 113 (63.8) | 111 (57.5) | 102 (55.7) | 103 (54.8) |  |
| Sex |  |  |  |  |  |  |
| Male | 397 (53.6) | 91 (51.4) | 98 (50.8) | 98 (53.6) | 110 (58.5) | 0.427 |
| Female | 344 (46.4) | 86 (48.6) | 95 (49.2) | 85 (46.4) | 78 (41.5) |  |
| Comorbidities |  |  |  |  |  |  |
| Hypertension | 248 (33.5) | 46 (26.0) | 66 (34.2) | 71 (38.8) | 65 (34.6) | 0.074 |
| Diabetes mellitus | 192 (25.9) | 36 (20.3) | 55 (28.5) | 54 (29.5) | 47 (25.0) | 0.183 |
| Dyslipidemia | 228 (30.8) | 31 (17.5) | 59 (30.6) | 72 (39.3) | 66 (35.1) | < 0.001 |
| Cerebrovascular accident | 103 (13.9) | 17 (9.6) | 35 (18.1) | 26 (14.2) | 25 (13.3) | 0.128 |
| Coronary artery disease | 138 (18.6) | 28 (15.8) | 33 (17.1) | 37 (20.2) | 40 (21.3) | 0.495 |
| Chronic kidney disease | 91 (12.3) | 18 (10.2) | 28 (14.5) | 24 (13.1) | 21 (11.2) | 0.582 |
| Bleeding history | 316 (42.6) | 62 (35.0) | 84 (43.5) | 77 (42.1) | 93 (49.5) | 0.049 |
| Degree of urbanization |  |  |  |  |  |  |
| Urban | 422 (57.0) | 94 (53.1) | 98 (50.8) | 109 (59.6) | 121 (64.4) | 0.143 |
| Suburban | 206 (27.8) | 51 (28.8) | 57 (29.5) | 52 (28.4) | 46 (24.5) |  |
| Rural | 69 (9.3) | 22 (12.4) | 21 (10.9) | 15 (8.2) | 11 (5.9) |  |
| Unknown | 44 (5.9) | 10 (5.6) | 17 (8.8) | 7 (3.8) | 10 (5.3) |  |
| Income level |  |  |  |  |  |  |
| Low income | 449 (60.6) | 124 (70.1) | 114 (59.1) | 104 (56.8) | 107 (56.9) | 0.002 |
| Median income | 177 (23.9) | 33 (18.6) | 46 (23.8) | 58 (31.7) | 40 (21.3) |  |
| High income | 102 (13.8) | 17 (9.6) | 26 (13.5) | 20 (10.9) | 39 (20.7) |  |
| Hospital ownership |  |  |  |  |  |  |
| Private | 445 (60.1) | 130 (73.4) | 125 (64.8) | 105 (57.4) | 85 (45.2) | < 0.001 |
| Public | 296 (39.9) | 47 (26.6) | 68 (35.2) | 78 (42.6) | 103 (54.8) |  |
| Hospital region |  |  |  |  |  |  |
| North | 364 (49.1) | 68 (38.4) | 75 (38.9) | 68 (37.2) | 153 (81.4) | < 0.001 |
| Middle | 240 (32.4) | 67 (37.9) | 76 (39.4) | 77 (42.1) | 20 (10.6) |  |
| South + East | 137 (18.5) | 42 (23.7) | 42 (21.8) | 38 (20.8) | 15 (8.0) |  |
| Medical center status |  |  |  |  |  |  |
| Non-medical center | 85 (11.5) | 67 (37.9) | 18 (9.3) | 0 (0.0) | 0 (0.0) | < 0.001 |
| Medical center | 656 (88.5) | 110 (62.1) | 175 (90.7) | 183 (100.0) | 188 (100.0) |  |
| Physician age |  |  |  |  |  |  |
| < 45 | 433 (58.4) | 118 (66.7) | 110 (57.0) | 99 (54.1) | 106 (56.4) | 0.077 |
| ≥ 45 | 308 (41.6) | 59 (33.3) | 83 (43.0) | 84 (45.9) | 82 (43.6) |  |
| Physician sex |  |  |  |  |  |  |
| Male | 641 (86.5) | 161 (91.0) | 168 (87.0) | 150 (82.0) | 162 (86.2) | 0.098 |
| Female | 100 (13.5) | 16 (9.0) | 25 (13.0) | 33 (18.0) | 26 (13.8) |  |
| Physician experience ≥ 5 years | 364 (49.1) | 29 (16.4) | 75 (38.9) | 123 (67.2) | 137 (72.9) | < 0.001 |

IQR, interquartile range
